# Dermal and inhalable cobalt exposure—Uptake of cobalt for workers at Swedish hard metal plants

**DOI:** 10.1371/journal.pone.0237100

**Published:** 2020-08-06

**Authors:** Fredrik Wahlqvist, Ing-Liss Bryngelsson, Håkan Westberg, Per Vihlborg, Lena Andersson

**Affiliations:** 1 Department of Occupational and Environmental Medicine, Faculty of Medicine and Health, Örebro University, Örebro, Sweden; 2 School of Medical Sciences, Faculty of Medicine and Health, Örebro University, Örebro, Sweden; 3 Inflammatory Response and Infection Susceptibility Centre (iRiSC), Faculty of Medicine and Health, Örebro University, Örebro, Sweden; University at Buffalo - The State University of New York, UNITED STATES

## Abstract

**Purpose:**

Cobalt exposure is known to cause adverse effects on health. A major use of cobalt is in the manufacture of hard metal. Exposure can lead to asthma, hard metal lung disease, contact allergy and increased risk of cancer. Cobalt is mainly absorbed from the pulmonary tract, however penetration through skin may occur. The relationships between exposure to inhalable cobalt in air and on skin and the uptake in blood and urine will be investigated, as well as the association between dermal symptoms and dermal exposure.

**Methods:**

Cobalt exposure in 71 workers in hard metal production facilities was measured as inhalable cobalt in the breathing zone and cobalt found on skin with acid wash. Uptake of cobalt was determined with concentrations in blood and urine. Correlations between exposure and uptake were analysed.

**Results:**

Inhalable cobalt in air and cobalt in blood and urine showed rank correlations with coefficients 0.40 and 0.25. Cobalt on skin and uptake in blood and urine presented correlation coefficients of 0.36 and 0.17. Multiple linear regression of cobalt in air and on skin with cobalt in blood showed regression coefficients with cobalt in blood (β = 203 p < 0.0010, and β = 0.010, p = 0.0040) and with cobalt in urine (β = 5779, p = 0.0010, and β = 0.10, p = 0.60).

**Conclusions:**

Our data presents statistically significant correlations between exposure to cobalt in air with uptake of cobalt in blood and urine. Cobalt on skin was statistically significant with cobalt in blood but not with urine.

## Introduction

Metals such as cobalt are elements naturally found in the environment. Cobalt can be found in air, water and tobacco smoke, though vegetables account for the majority of human intake [1,2]. Cobalt is essential as a metal constituent of vitamin B12 [2–4] but its toxic potential from excessive exposure is well known [5,6]. Cobalt is used in several different areas such as the manufacture of paint, making of jewellery [7] and metal prostheses in healthcare [8], though the major use of cobalt is in the production of metal alloys such as superalloys, magnetic alloys, high-strength steels and hard metal [9]. Hard metal is produced in a sintering process where cobalt is mixed with divided tungsten carbide [10].

Humans normally contain low levels of cobalt in tissue and fluids, however occupational exposures may increase the levels [1]. Exposure occurs mainly through air, food and drink [2,4]. In occupational exposure, the source of cobalt exposure is primarily through inhalation. Exposure to cobalt has been associated with causing inflammations in the higher respiratory tract, such as rhinitis and bronchitis [11,12], as well as diseases concerning the lower respiratory tract. Asthma can be induced by occupational exposure to cobalt and cobalt can cause fibrotic changes in lung tissue [13–15]. With high levels of cobalt in the air, pneumoconiosis has been observed [16,17], while obstructive pulmonary disease is observed at lower levels [4]. Development of obstructive lung diseases may be increased by chronic exposure to cobalt [18].

Association has been shown between cobalt and the development of contact allergy and eczema through direct contact with the skin as well as airborne particles [19–21]. A significant increase in risk of lung cancer in hard metal workers exposed to cobalt was shown in a group of studies [22–24]. Cobalt in combination with tungsten carbide is classified as likely carcinogenic to humans [9]. Cobalt has formerly been used as an additive in beer in Canada, the USA and Belgium, causing pericardial effusion, elevated haemoglobin and congestive heart failure in some high consumers [3,4,25], and is classed as cardiotoxic [1]. Effects on other organs have been shown to be associated to cobalt exposure, such as impaired thyroid function [3,26]. Orthopaedic implants containing cobalt have been associated with increase of cobalt concentrations in blood and urine [27–29] and higher incidence of malignant tumours, with sarcomas and other malignant tumours observed around the locations of the implants [1].

Cobalt exposure is of importance since it affects a large population of workers. Sweden is one of the dominant producers of hard metal, where approximately 11 000 people may be in direct contact with cobalt and about 20 000 welders may be exposed to cobalt. In 2007 377 tonnes of cobalt and cobalt compounds were handled in Sweden [10]. The occupational exposure to cobalt can be assessed with biological monitoring and measured air levels [30–32]. Occupational exposure to cobalt through inhalation has been acknowledged for decades [33], and penetration through skin from contact with materials containing cobalt or airborne particles containing cobalt has been demonstrated [34–36]. Studies have found that through absorption skin and the gastrointestinal tract participate in the uptake of cobalt in urine in occupationally exposed individuals [34,37,38]. Previous research has shown associations between airborne exposure to cobalt with concentrations of cobalt in blood and urine [31,32,39]. Here we present correlations between cobalt exposure in air and on skin with uptake in blood and urine as well as correlations between dermal exposure and symptoms.

## Methods

### Study group

The study was performed at two Swedish hard metal industries, employing 130 and 1 400 workers, and with a production during the measurements days of 18 500–44 000 items and 1 484 000–1 542 000 items of hard metal products. The hard metal details produced are based on wolfram carbide where cobalt is used as binder. The companies produce smaller details of cemented carbide materials for cutting work and rock drilling, of 10–250 gram/piece and larger products for the car and plane industry of 10–50 kg/piece.

The departments included were forming (prototype), lab, pressing, powder and maintenance. Pressing and powder department represented the majority of the workers. A total of 71 hard metal workers, representing mainly dayshift, participated in this study, comprising 62 men and 9 women. The mean age was 42 years, ranging from 20 to 65, a majority of the workers were between 30 to 60 years, some 22% under the age of 30 and only 9% above 60 years old. They had worked in the same workplace for 11 years on average, ranging up to 45 years of employment. In total 62 participants reported their smoking habits: 2 subjects were current smokers, 12 ex-smokers and 48 had never smoked (Table 1).

**Table 1:** Base characteristic for the study population (n = 71)^a^.

		n	%
Sex	Men	62	87
	Women	9	13
Age	<30	16	22
	30–59	49	69
	≥60	6	9
Years at current workplace	<5	22	31
	5–9	16	22
	≥10	26	37
Years at current work tasks	<5	26	37
	5–9	19	27
	≥10	18	25
Smoking habits	Non-smoker	48	68
	Ex-smoker	12	17
	Current smoker	2	3

n: number of participants

^a^: Not all participants provided information regarding work history and smoking habits

Persons with eczema are generally not recruited into positions associated with elevated exposure to hard metal or cobalt. If eczema is detected during employment, there will be evaluation for reassignment.

### Study design

Measurements were performed from March 2017 to October 2018 in two hard metal production facilities in Sweden, and forms a cross-sectional study portion of a major observation study on particle-induced inflammation. The workers received information about the project and written consent was obtained prior to participation. The study was approved by the Regional Ethical Review Board, Uppsala (Dnr. 2017/050), including the informed consent procedures.

A general questionnaire was completed by all participants, containing items about current and previous working conditions, height, weight, smoking habits, physical activity and dermal diseases. Initially 74 workers from the hard metal plants exposed to cobalt were invited to participate. Due to illness and pregnancy, two workers did not fulfil the measurements. Furthermore, one person was excluded from the analysis due to accounted for abnormal values from an accident in the workplace, which did not represent normal working conditions.

The investigation comprised air sampling of inhalable dust and cobalt and biological sampling of blood, urine and skin. The air sampling of inhalable dust and cobalt was performed after a work-free weekend on the morning shift (6 am-2 pm) at the 2^nd^ or 3^rd^ working day in the week. Blood sampling was performed the same day as the air sampling, before and after the work shift and followed up after two days, i.e., after shift the 4^th^ or 5^th^ day of the working week. The same day as the air sampling during the work shift the workers skin exposure to cobalt was measured during 2 h. The pre- and post-shift sampling design aimed to control for circadian variation when comparisons were made, evaluating both long-term effects, effects over shift and difference between different days in the week. All participants were at work during our investigation.

### Aerosol measurements and cobalt analysis

Inhalable cobalt exposure was determined through measurements in the breathing zone for all workers during a work shift after a work-free weekend on the morning shift (6 am-2 pm) at the 2^nd^ or 3^rd^ working day in the week. Inhalable dust was collected by a GSP-sampler with a 37 mm filter of 3 μm pore-size and with an airflow of 3.5 l/min. [40]. The dust concentration was analysed gravimetrically and for determination of cobalt in the dust, filters and dust were dissolved with acid and analysed using inductively coupled plasma mass spectrometry (ICP-MS) [41].

### Skin exposure

Skin exposure was measured during 2 h of the same work shift the workers air exposure to cobalt was measured. Prior to measurement, participants washed their hands with soap and water and an established method of washing containing a concentration of 0.1% HNO_3_ was used for the chosen areas of the skin. Time of day varied for participants though all exposures lasted for two hours. The workers were asked to do their work as usual, using the same protective equipment including gloves, and were asked not to wash their hands during this time. After exposure the areas of interest were washed with wipes containing the acid and placed in vials. Samples were taken from the back of the hand, the palm and the first four fingers on the dominant hand and the fifth finger on the non-dominant hand. The fifth finger on the dominant hand was covered to act as a blank sample. To solubilise the wipes from contamination, the vials were mixed with a liquid containing 1% HNO_3_. The fluid in the vials was then analysed for cobalt using ICP-MS [41].

### Biological monitoring

Blood and urine samples were collected before and after the work shift on the same day that air and skin exposure to cobalt was measured, to represent the concentration for the test day, i.e., after a work-free weekend on the morning shift (6 am-2 pm) at the 2^nd^ or 3^rd^ working day in the week. After two days another biological sampling was undertaken at the end of the day, i.e., after shift the 4^th^ or 5^th^ day of the working week. Specific weights of the urine samples were measured using a refractometer, and a corrected value was calculated. Blood and urine samples were analysed using ICP-MS to determine the content of cobalt [41]. As controls in the analyses, certified Seronorm with known amounts of cobalt for blood and urine were used. All analysis was performed at the Department of Occupational and Environmental Medicine at the Örebro University Hospital. The study design is illustrated in S1 Fig.

### Exposure measures

Exposure was presented as 8-hour time weighted averages (TWA, mg/m^3^) of inhalable cobalt, skin exposure as μg/cm^2^/2h cobalt and the uptake of cobalt in blood and urine as nmol/l. For the established high exposure jobs respirators were worn, in particular in the powder department and when changing powder at the pressing department. However, for the 26 subjects using respirators, we adjusted our personal measurements for the use of respirators, aiming to reflect true exposures. We based the adjustments for users of respirators on the assumption of no exposure for the time spent using the mask, and the corresponding background air concentrations for the rest of the shift, creating an 8-hour TWA shift [42].

The participants were organised in groups according to which department they were located in. Respiratory protective equipment (P3-filtering face masks) were used by workers presumed to be exposed to higher levels of dust and cobalt, especially those in the powder department and in the pressing department when changing powder.

### Occupational exposure limits

The Swedish Work Environment Authority (SWEA) has defined Occupational Exposure Limits (OEL) for air pollution, defined by the maximum acceptable value for a work day of eight hours. For cobalt in inhalable dust, the SWEA OEL is 0.02 mg/m^3^ [43]. The American Conference of Governmental Industrial Hygienists (ACGIH) has established 0.02 mg/m^3^ for cobalt as the 8-hour time-weighted average (TWA) [44]. The SWEA has no limit values for cobalt in blood or urine though the Department of Occupational and Environmental Medicine at Örebro University Hospital, Sweden, considers values of 10 nmol/l for both blood and urine as normal compared to unexposed referents [45]. The Finnish Institute of Occupational Health (FIOH) has normal values for cobalt in blood and urine of 14 nmol/l and 25 nmol/l, respectively, with a limit value for cobalt in urine of 130 nmol/l [46]. The ACGIH has determined a Biological Exposure Index (BEI) for cobalt in urine of 255 nmol/l [47]. The dermal limit value has yet to be established.

### Statistical methods

The study population was presented with descriptive statistics including factors such as sex, age, BMI, years at workplace, years at current work tasks and smoking habits. For descriptive purposes, the aerosol concentrations of inhalable cobalt are presented by job title as 8-hour TWAs for the full workday. Data are presented both as unadjusted and adjusted for the use of respirators. Standard parameters such as arithmetic mean (AM), standard deviation (SD), geometric mean (GM), geometric standard deviation (GSD) and range were calculated for the log normal distribution of all the measurements (Tables 2 and 3). A corresponding description, mean and range, of the blood, urine and skin concentration by sampling day is also presented (Table 4).

**Table 2:** Exposure concentration levels of inhalable cobalt by job title, non-adjusted for the use of respirators.

	Inhalable cobalt (mg/m^3^)							
Job title	n	AM	Median	SD	GM	GSD	Min	Max
Forming/prototype	5	0.0020	0.0017	0.0012	0.0017	2.0	0.00061	0.0034
Lab	5	0.00060	0.00037	0.00048	0.00046	2.3	0.00020	0.0013
Maintenance	7	0.00099	0.00092	0.00047	0.00090	1.6	0.00039	0.0018
Powder	15	0.0071	0.0057	0.0044	0.0059	1.9	0.0018	0.019
Pressing	39	0.0028	0.0022	0.0026	0.0018	2.8	0.00016	0.010
Total	71	0.0033	0.0022	0.0035	0.0020	3.0	0.00016	0.019

n: number of measurements; AM: arithmetic mean; SD: standard deviation; GM: geometric mean; GSD: geometric standard deviation

**Table 3:** Exposure concentration levels of inhalable cobalt by job title, adjusted for the use of respirators.

	Inhalable cobalt (mg/m^3^)							
Job title	n	AM	Median	SD	GM	GSD	Min	Max
Forming/prototype	5	0.00094	0.00061	0.00050	0.00084	1.6	0.00058	0.0017
Lab	5	0.00060	0.00037	0.00048	0.00046	2.3	0.00020	0.0013
Maintenance	7	0.00089	0.00078	0.00056	0.00073	2.1	0.00022	0.0018
Powder	15	0.0022	0.00090	0.0030	0.00084	4.5	0.00010	0.0092
Pressing	39	0.0019	0.0011	0.0023	0.0011	2.8	0.00016	0.010
Total	71	0.0017	0.00090	0.0022	0.00092	3.0	0.00010	0.010

n: number of measurements; AM: arithmetic mean; SD: standard deviation; GM: geometric mean; GSD: geometric standard deviation

**Table 4:** Concentration levels cobalt in blood and urine and cobalt on skin by job title.

	Department	n	Mean	Median	Min	Max	SD
**Blood cobalt,**	Forming/prototype	5	4.2	4.8	2.8	5.1	0.98
**before shift**	Lab	5	5.4	4.7	2.8	11	3.4
(nmol/l)	Pressing	39	5.8	5.6	2.8	9.5	1.7
	Powder	15	8.1	8.2	4.7	11	1.9
	Maintenance	7	6.7	6.5	2.8	11	2.9
	Total	71	6.2	5.7	2.8	11	2.2
**Blood cobalt,**	Forming/prototype	4	4.1	3.8	3.5	5.2	0.78
**after shift day 1**	Lab	5	5.4	4.7	2.8	11	3.4
(nmol/l)	Pressing	38	6.3	5.6	3.5	13	2.2
	Powder	15	9.6	9.2	5.6	14	2.7
	Maintenance	7	7	7	2.8	10	2.8
	Total	69	6.9	6.5	2.8	14	2.8
**Blood cobalt,**	Forming/prototype	5	4.3	4.5	3.5	5.2	0.77
**after shift day 3**	Lab	5	5.3	4.3	2.8	11	3.4
(nmol/l)	Pressing	37	6.4	5.8	3.5	15	2.3
	Powder	12	8.7	8.8	5	12	2.2
	Maintenance	7	7.1	6.5	2.8	11	2.9
	Total	66	6.6	5.9	2.8	15	2.6
**Urine cobalt,**	Forming/prototype	5	14	14	4.9	25	7.5
**before shift**	Lab	5	27	9.7	4.1	91	37
(nmol/l)	Pressing	39	29	24	4.5	90	21
	Powder	15	52	31	8.5	250	61
	Maintenance	7	41	38	3.9	85	24
	Total	71	34	25	3.9	250	35
**Urine cobalt,**	Forming/prototype	5	21	16	4.9	53	19
**after shift day 1**	Lab	5	18	17	3.4	31	13
(nmol/l)	Pressing	38	37	28	6.6	99	24
	Powder	15	81	63	12	220	59
	Maintenance	7	40	36	16	77	25
	Total	70	44	35	3.4	220	39
**Urine cobalt,**	Forming/prototype	5	16	10	4.9	44	16
**after shift day 3**	Lab	5	13	5,2	3.4	42	16
(nmol/l)	Pressing	38	46	38	7.5	120	31
	Powder	12	55	46	15	120	37
	Maintenance	7	53	30	5.4	150	53
	Total	67	44	33	3.4	150	35
**Skin cobalt**	Forming/prototype	5	3.5	3.6	0.62	8.8	3.3
(μg/cm^2^/2h)	Lab	5	6.5	1.9	0.99	26	11
	Pressing	39	24	7.5	0.1	200	47
	Powder	15	10	7.5	0.67	29	8.5
	Maintenance	7	30	6.9	1.9	170	61
	Total	71	19	6.8	0.1	200	40

n: number of participants; SD: standard deviation

The content of cobalt in an 8-hour TWA air concentration is presented as mg/m^3^. The various measured areas of the skin were added to represent one total skin exposure and is presented as μg/cm^2^/2h. Cobalt in blood and urine is presented as nmol/l. Analytical statistics were performed on the individual measurements of all the samples. Correlation coefficients were calculated with Spearman's rho test between air, skin, blood and urine. Linear regression was used to analyse the relationships between two variables and multiple linear regression analysis was used to look at one dependent variable toward two independent variables. A p-value of less than 0.05 was considered as statistically significant. Software used for analysis and compilation of data was SPSS 25.0.

## Results

The study included 71 participants, consisting of more men than women, who all worked in the hard metal industry (Table 1). Most were older than 30 and younger than 60 years. Average age was 42 ± 12.5 years. There was a variation regarding how long they had worked in the workplace and how long they were assigned to their current tasks. Relatively few stated that they are current smokers or previously smoked, with almost 70% stating that they have never smoked.

Personal air exposure measurements presented values for workers in all departments. The amount of cobalt varied between 0.00010–0.010 mg/m^3^ for samples adjusted for the use of respirators and 0.00016–0.019 for non-adjusted samples (Tables 2 and 3). No worker had levels exceeding the Swedish OEL (0.02 mg/m^3^). The total average exposure concentration levels of inhalable cobalt decreases when adjusting for the use of respirators, from 0.0033 mg/m^3^ to 0.0017 mg/m^3^. The highest decrease is in the powder department, from 0.0071 mg/m^3^ to 0.0022 mg/m^3^.

Concentrations of cobalt in blood showed a mean of 6.2 nmol/l before work shift, 6.9 nmol/l after shift and 6.6 nmol/l two days later (Table 4). Of all samples, the highest levels were found in the powder department (mean 9.6 nmol/l after shift, day one), with nine samples (4%) exceeding normal values set by the Department of Occupational and Environmental Medicine at Örebro University Hospital, Sweden, as well as normal values set by the FIOH (10 and 14 nmol/l).

Levels in urine presented a mean of 34 nmol/l before shift, 44 nmol/l after shift and 44 nmol/l after two days, with 101 samples (49%) exceeding normal value set by the FIOH (25 mmol/l) and 181 samples (87%) exceeding normal value set by the SWEA (10 nmol/l). Urine concentrations exceeding the FIOH limit value (130 nmol/l) were found in four samples (2%) from four workers. All four of them worked in the powder department and used respiratory protective equipment during the work shift when higher levels of cobalt and dust were expected. No worker exceeded the BEI for cobalt in urine (255 nmol/l). Concentrations of cobalt on skin varied between 0.1–200 μg/cm^2^/2h. The highest concentrations were measured in workers in the maintenance (mean 30 μg/cm^2^/2h) and pressing (mean 24 μg/cm^2^/2h) departments.

From the questionnaire, the prevalence of skin symptoms among hard metal workers showed that 42% had dry skin, 14% had eczema as a child, 7% have experienced hand eczema, 6% have experienced face eczema and 6% had eczema on their forearms (Table 5).

**Table 5:** Prevalence of skin symptoms among hard metal workers and referents, percentage of answers.

Skin symptom	Yes n %		No n %		Missing cases
Eczema as a child?	10	14	60	85	1
Dry skin?	30	42	39	55	2
Ever experienced hand eczema?	5	7	66	93	0
If yes:					
Diagnosed hand eczema?	2	3			
Dermally tested?	3	4			
Eczema on the forearms?	4	6	60	85	7
If yes:					
Have been tested due to eczema on the forearms?	1	1			
Ever experienced face eczema?	4	6	65	92	2
If yes:					
Diagnosed face eczema?	0	0			
Tested for the face eczema?	1	1			

n: number of participants

The rank correlation coefficients for inhalable cobalt and cobalt in blood, urine and on skin (Table 6) were all statistically significant, except for skin with urine. Cobalt in blood and urine showed the greatest correlation with a coefficient of 0.44.

**Table 6:** Rank correlation coefficients between inhalable cobalt adjusted for the use of respirators and cobalt in blood, urine and on skin.

	Inhalable cobalt	Skin cobalt	Urine cobalt
**Blood cobalt**	0.40**	0.36**	0.44**
**Inhalable cobalt**	-	0.26*	0.25**
**Skin cobalt**	-	-	0.17

*: Correlation is significant at the 0.05 level (two-tailed)

**: Correlation is significant at the 0.01 level (two-tailed)

Relationships between exposure and uptake of cobalt were examined by linear regression (Table 7). Simple linear regression show statistical significant results for cobalt in blood and inhalable cobalt (β = 199, p = 0.0010) and cobalt in blood and skin (β = 0.010, p = 0.0030). Cobalt in urine and inhalable cobalt showed a statistically significant correlation (β = 5759, p = 0.0010) but not urine and skin (β = 0.040, p = 0.65). Using multiple regression analysis both exposure factors were analysed; for uptake in blood and urine the regression coefficients were higher. For cobalt in air and on skin with cobalt in blood (β = 203 p < 0.0010, and β = 0.010, p = 0.0040) and with cobalt in urine (β = 5779, p = 0.0010, and β = 0.10, p = 0.60) they were statistically significant, except for exposure on skin and uptake in urine. For the blood analyses, the difference in the biological samples before and after work shift was used.

**Table 7:** Linear and multiple linear regression analysis between exposure, inhalable cobalt adjusted for the use of respirators and cobalt on skin, and uptake of cobalt in blood and urine.

Analysis	Uptake	Exposure	n	r^2^	β	95% CI	p-value
Simple linear	Blood cobalt	Inhalable cobalt	69	0.16	199	89–310	0.0010
regression		Skin cobalt	69	0.12	0.010	0.0030–0.016	0.0030
	Urine cobalt	Inhalable cobalt	70	0.15	5759	2392–9126	0.0010
		Skin cobalt	70	0.0030	0.040	-0.16–0.25	0.65
Multiple linear	Blood cobalt	Inhalable cobalt	69	0.29	203	101–306	<0.0010
regression		Skin cobalt			0.010	0.0040–0.020	0.0040
	Urine cobalt	Inhalable cobalt	70	0.15	5779	2392–9165	0.0010
		Skin cobalt			0.10	-0.14–0.24	0.60

n: number of samples; r^2^: explained variance; β: regression coefficient; CI: confidence interval

To visualize the data in the study, scatter plots for the exposure to cobalt on skin or cobalt as inhalable air, adjusted or non-adjusted for the use of respirators, compared to measured cobalt in blood or urine is presented in S1 Appendix.

## Discussion

This study examined the relationships between inhalable cobalt in air, cobalt on skin and cobalt in blood and urine. The results showed statistically significant correlations between inhalable cobalt and cobalt in blood and urine, as presented with Spearman’s rho test and simple and multiple linear regression analyses. The statistically significant correlation in the regression analysis for skin exposure with cobalt in blood confirms skin as a pathway for cobalt uptake in blood. A prior Finnish study concluded that skin exposure is an important route for cobalt uptake in continuous exposure, though not to the same extent as inhalable exposure [38], while a study in Swedish hard metal plants suggest skin exposure to cobalt could affect the total uptake at the same order of magnitude as air exposure [48]. Levels of cobalt in the breathing zone in the Finnish study were in line with levels measured in the Swedish study, ranging between 0.00016–0.019 mg/m^3^ and 0.000028–0.056 mg/m^3^ [49]. Amount of cobalt in air varied between the departments: the highest levels were found in the powder and pressing departments, which corresponds with findings from earlier studies [18,49].

This research is unique as it investigates skin exposure to cobalt as well as urine as an indicator for uptake in the body in the hard metal production industry. Cobalt in skin has no limit value, which is why it is interesting to include, in order to examine its importance for the uptake. Levels in skin showed a total mean of 19 μg/cm^2^/2h and range of 0.1–200 μg/cm^2^/2h. The study by Klasson showed a total mean of 12 μg/cm^2^/h and range of 0.046–100 μg/cm^2^/h, but this reflects a 1-hour exposure time. Maintenance had the highest cobalt levels found on skin with a mean of 30 μg/cm^2^/2h, whilst in the aforementioned study the highest levels were found in the pressing department (23 μg/cm^2^/h) [49]. A previous Swedish study, investigating skin exposure to cobalt in the production of gas turbines and space propulsion components, found levels within the range of 0.0013–4.5 μg/cm^2^/2h [36].

Results from biological monitoring show an uptake of cobalt with levels exceeding normal values set for unexposed people. Cobalt in blood showed lower levels in this study than past studies [44], with a total mean of 6.9 nmol/l and range of 2.8–15 nmol/l compared to 7.9 nmol/l and 3.2–110 nmol/l. No value exceeded the BEI, though 4% of the samples exceeded normal values set by the Department of Occupational and Environmental Medicine at Örebro University Hospital, Sweden, and FIOH. This implies that workers were exposed to cobalt to a higher extent than unexposed people. Measured levels of cobalt in blood for the hard metal workers in this study are below measured levels in blood in a study of metal hip toxicity for 251 hip implant patients and in a study for metal-on-metal (MOM) hip implants patients with mean levels of 43 and 112 nmol/l, respectively [28,29]. The cobalt levels in this study is far below the safety level for systemic effects of 1700 nmol/l (100μg/l respecting a safety factor of 3) [50].

Urine levels of cobalt in this study varied between 3.4–250 nmol/l, with a mean value of 34 nmol/l before shift and 44 nmol/l after shift. This is lower than an earlier study investigating cobalt in urine, which found a mean of 241 nmol/l and range of 8–2705 nmol/l [38]. The FIOH limit value was exceeded by 2% of the samples in the powder department. These workers used protective respiratory equipment. Companies require the use of respiratory protective equipment when exposure to higher levels of dust and particles is presumed. Workers protect themselves against exposure though still have urine levels above the FIOH limit value, which could indicate that other factors such as exposure to skin are important.

Levels of airborne cobalt in the workplace have decreased considerably over the past years, which has been associated with improved hygiene and protective measures [51]. This decrease can be illustrated by the current as well as recent studies [49,52]. Since the air levels in the facilities are decreasing, it is interesting to investigate the importance of dermal exposure. All measured levels of cobalt in the breathing zone are below the OEL (0.02 mg/m^3^). However, blood and urine show relatively high concentrations of cobalt. It is known that penetration of cobalt through the skin may occur [34–36]. Skin exposure was in line with earlier studies, which may explain skin as an entry for cobalt to the body.

Workers with presumed high exposure to cobalt dust use respiratory protection equipment (P3-filtering face masks), which decrease their inhalable exposure even more. In this study we present cobalt exposure both non-adjusted and adjusted for the use of respirators by the assumption of no exposure for the time spent using the mask. The correction have a limitation since respiratory protection in practice might not provide 100% efficiency.

As mentioned in the introduction, several systemic effects including the respiratory system, cardiovascular effects, lung cancer and skin diseases could occur at high cobalt exposure levels. Neither the air nor the dermal exposures expressed as uptake of cobalt in blood or urine seem sufficient to cause any of these major systemic health effects.

One more possible entry for cobalt is via ingestion. Unintentional ingestion can occur at lunch-time via poorly washed hands or cobalt on other places of the skin and clothes contaminating food. Another means is hand to mouth contact which can occur beyond food intake at any time at the workplace. Use of snuff could be one reason for this movement and oral exposure. This could explain the findings in our study of high concentrations in uptake of cobalt when levels of inhalable cobalt are low. Only 3% of the workers in our study had a diagnosis of hand eczema. The diagnosis should result in no employment within the companies. It is plausible that impaired skin barriers in the form of eczema could be a possible explanation for increased penetration of cobalt.

By examining the different areas of the skin chosen for measurement, this research can distinguish the back of the hand as the least exposed area. This is confirmed by earlier findings from the Department of Occupational and Environmental Medicine examining cobalt in other industries in Sweden, indicating that skin exposure could primarily result from contact with solid items, rather than airborne particles. The difference between cobalt found on skin surfaces in the participants could be explained by the fact that the workers had varying work tasks during the exposure period and that the use of gloves varied, as did the condition of the gloves. This corresponds with findings from a previous study [36].

Methods used for measurement in our study are well established and are based on recommendations and experiences from prior studies and scientific literature, as well as practical conditions. The acid wash technique for skin exposure is a non-invasive method suitable for measurements in the industrial environment [53]. Areas of interest for measurement were zones with the highest exposure according to experience from similar studies. Samples were gathered from the hand and thus does not consider total body surface. Our measured skin exposures represent 2-hour exposure and do not reflect the entire work shift. It is therefore not possible to conclude how skin exposure occurred for the other six hours of the day, such as the participants’ exposure from air. What it can however distinguish is that this represents normal activity for the workers for this period, and when taking toilet visits and lunch break into account this is assumed to be in line with exposure of eight hours, with some reservations.

The workers had not been released from work prior to measurement. It is possible that this could lead to higher concentrations of cobalt from the first measurement. Experience from similar studies have shown that it did not matter significantly whether they were away from work for a few days prior or not. This is believed to be because these individuals are exposed to cobalt incessantly and have chronic levels of cobalt.

Data in this study presents urine as an indicator for uptake from cobalt exposure, though is not correlated to exposure on skin, whilst correlation appeared between cobalt via airways and urine. The same discovery was found in an earlier study [38]. One explanation could be that it takes more time for cobalt to penetrate the skin and travel from blood to urine than to travel from airways to eventually establish in urine. It is possible that urine samples later in the evening or before shift the next day as a follow-up test could have led to better compliance in the uptake of cobalt in urine. Also the distribution time for cobalt particles from air and skin to blood has a certain lag time and the blood sample measurements may better represent the workday exposure if taken later in the evening, this is though harder to perform since the workers leave the work facility in the end of the workday. This lag might contribute somewhat to the relatively low amount of variance explained by the model.

Results in this study can contribute to increased knowledge about exposure and uptake of cobalt and motivate changes to reduce this, and thereby reducing the risks of causing various diseases and cancer. The data can be of significance for the reevaluation of hygienic and biological limit values. This is important to examine because it affects a large population of workers. By collecting data from different departments as well as individual data this study can deliver reports to the corporations which can result in positive changes in the work environment. Levels of cobalt in air are low at the facilities. Nevertheless, levels of cobalt in workers’ blood and urine exceed normal values for unexposed persons. Skin exposure may be a source of this elevation. The companies are recommended to continue working on hygiene routines and the use of protective gear such as gloves and respiratory protective equipment in order to decrease employees' individual uptake.

## Conclusion

Statistically significant correlations appeared between exposure to cobalt in air with uptake of cobalt in blood and urine. Exposure to cobalt on skin showed significant correlation with uptake in blood but not urine. Our results show skin as a pathway, through the skin or by ingestion, to uptake of cobalt in the blood. This data also presents urine as an indicator for uptake from cobalt exposure, though is not correlated to exposure on skin. Further studies are required to explore the relationships between exposure and uptake and the importance of dermal exposure.

## Supporting information

S1 Fig Flow chart of the study's procedure. (PDF)

S1 Appendix Scatter plots of exposure to cobalt on skin or cobalt as inhalable air, adjusted or non-adjusted for the use of respirators, compared to measured cobalt in blood or urine. (PDF)
