# Supplementary figures and images for: Dermal and inhalable cobalt exposure—Uptake of cobalt for workers at Swedish hard metal plants
Source: PLoS One. 2020 Aug 6;15(8):e0237100. doi: 10.1371/journal.pone.0237100 (PMC7410254; doi:10.1371/journal.pone.0237100)

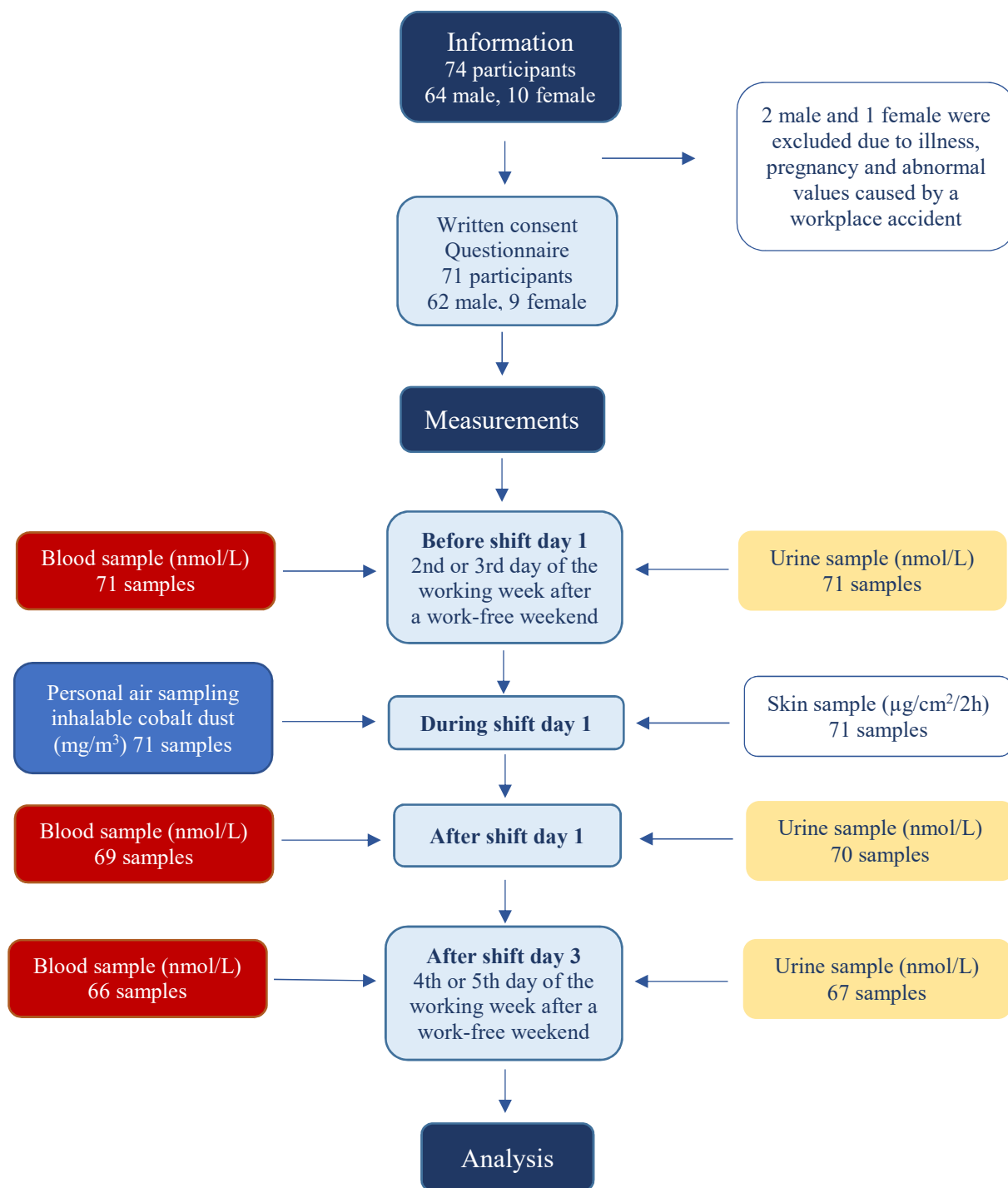

**S1 Fig. Flow chart of the procedure of the study.**

Supplement: S1 Fig — (PDF) [file pone.0237100.s001.pdf]
