# Supplementary material for: Dermal and inhalable cobalt exposure—Uptake of cobalt for workers at Swedish hard metal plants
Source: PLoS One. 2020 Aug 6;15(8):e0237100. doi: 10.1371/journal.pone.0237100 (PMC7410254; doi:10.1371/journal.pone.0237100)
Supplement: S1 Appendix — (PDF) [file pone.0237100.s002.pdf]

Appendix. Scatter plots of exposure to cobalt on skin or cobalt as inhalable air, adjusted or non-adjusted for the use of respirators, compared to measured cobalt in blood or urine.

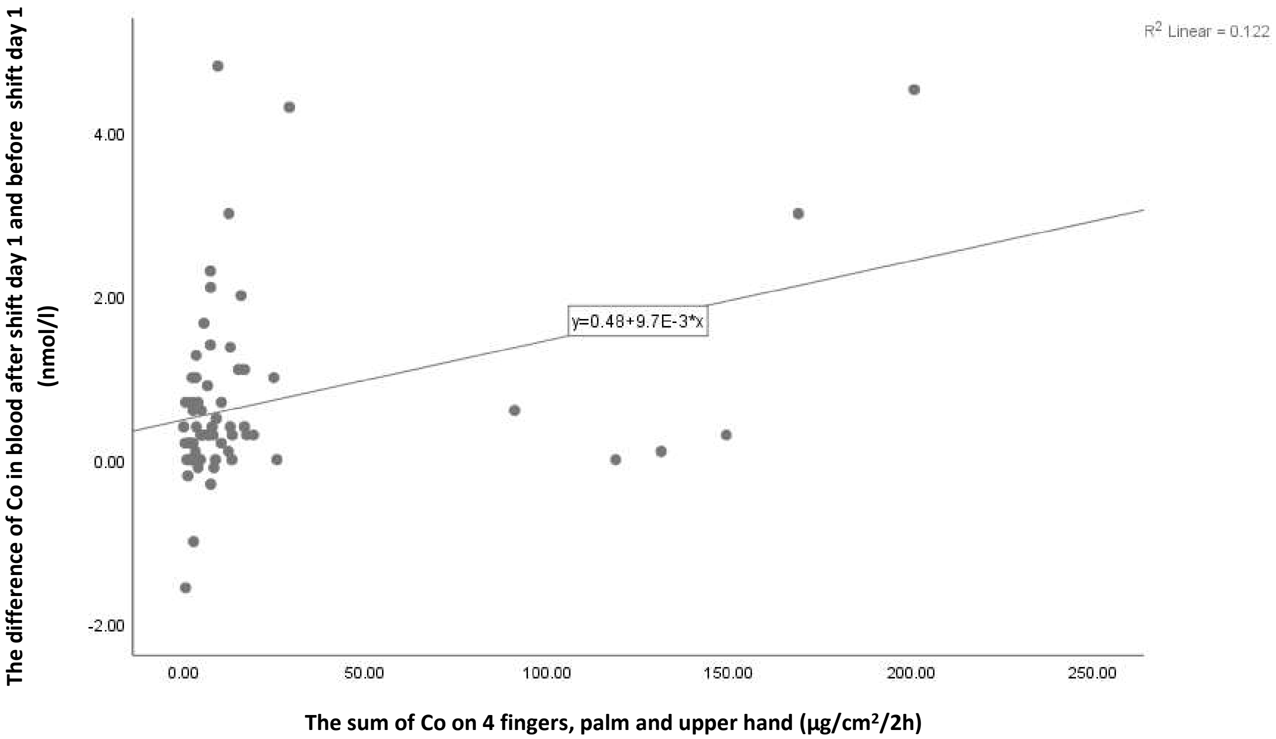

Fig 2. Scatter plot for Co on skin compared to Co in blood.

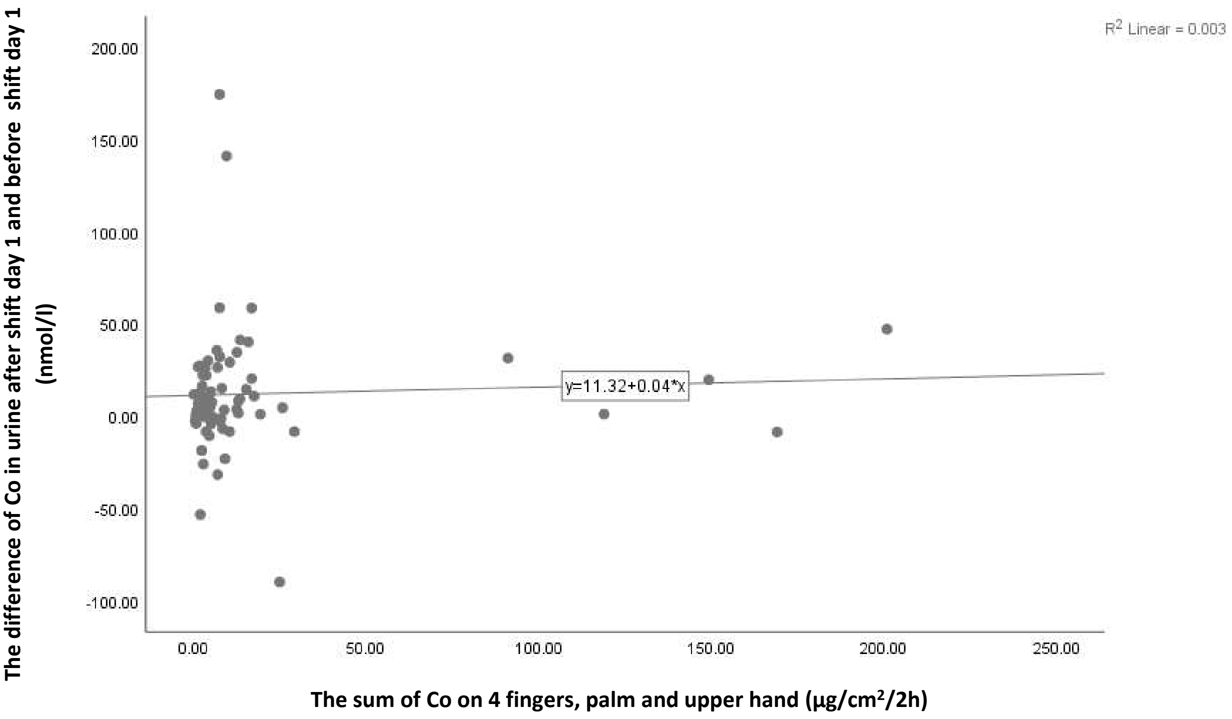

Fig 3. Scatter plot for Co on skin compared to Co in urine.

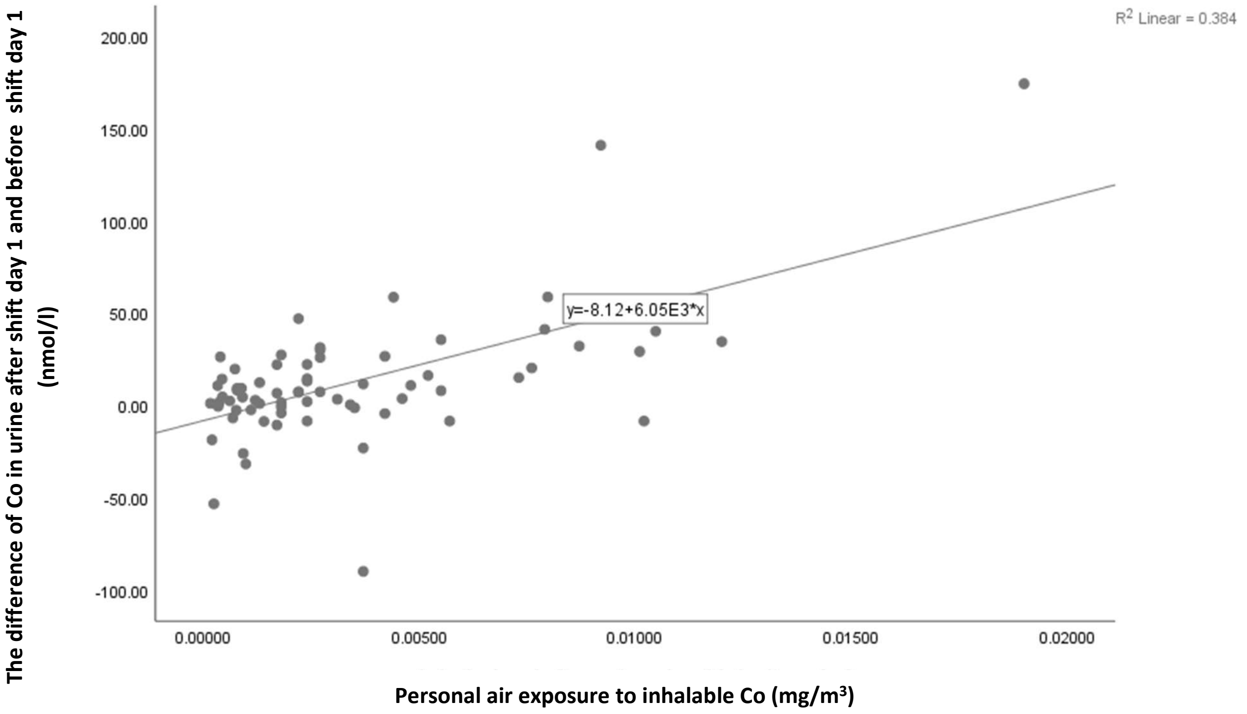

Fig 4. Scatter plot for inhalable Co compared to Co in urine.

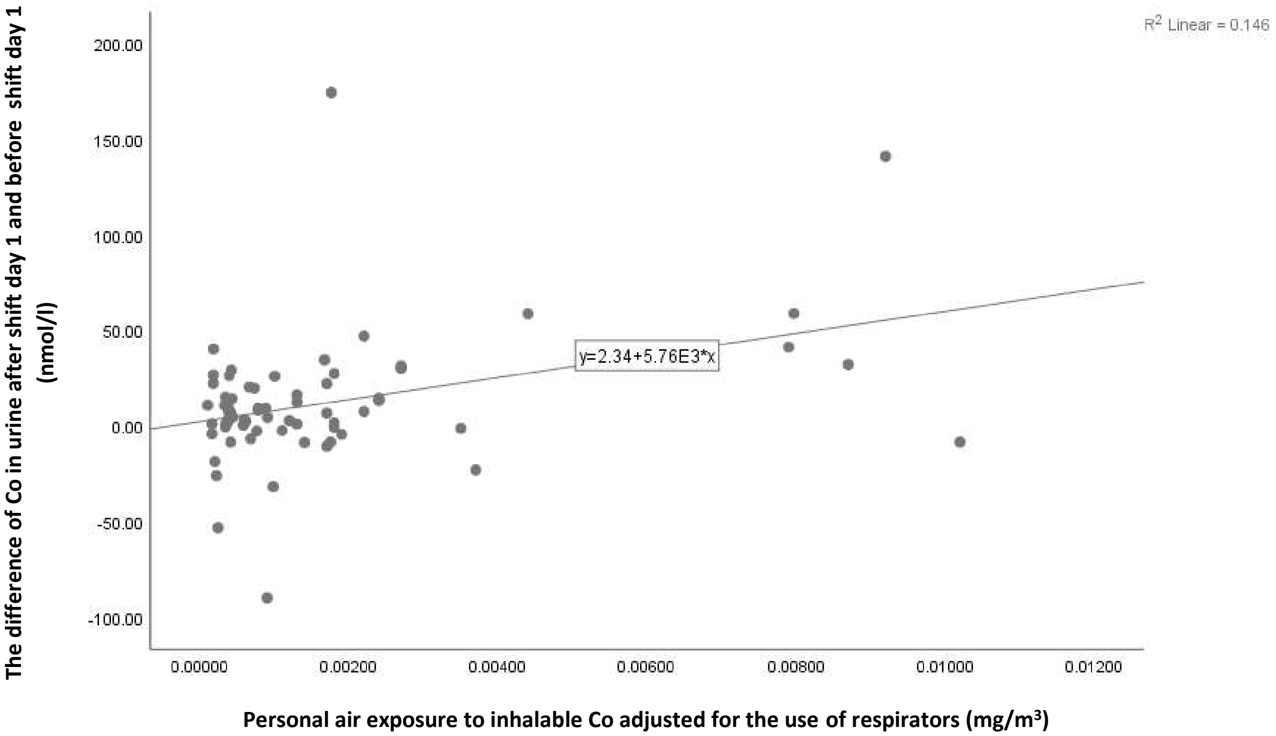

Fig 5. Scatter plot for inhalable Co adjusted for the use of respirators compared to Co in urine.

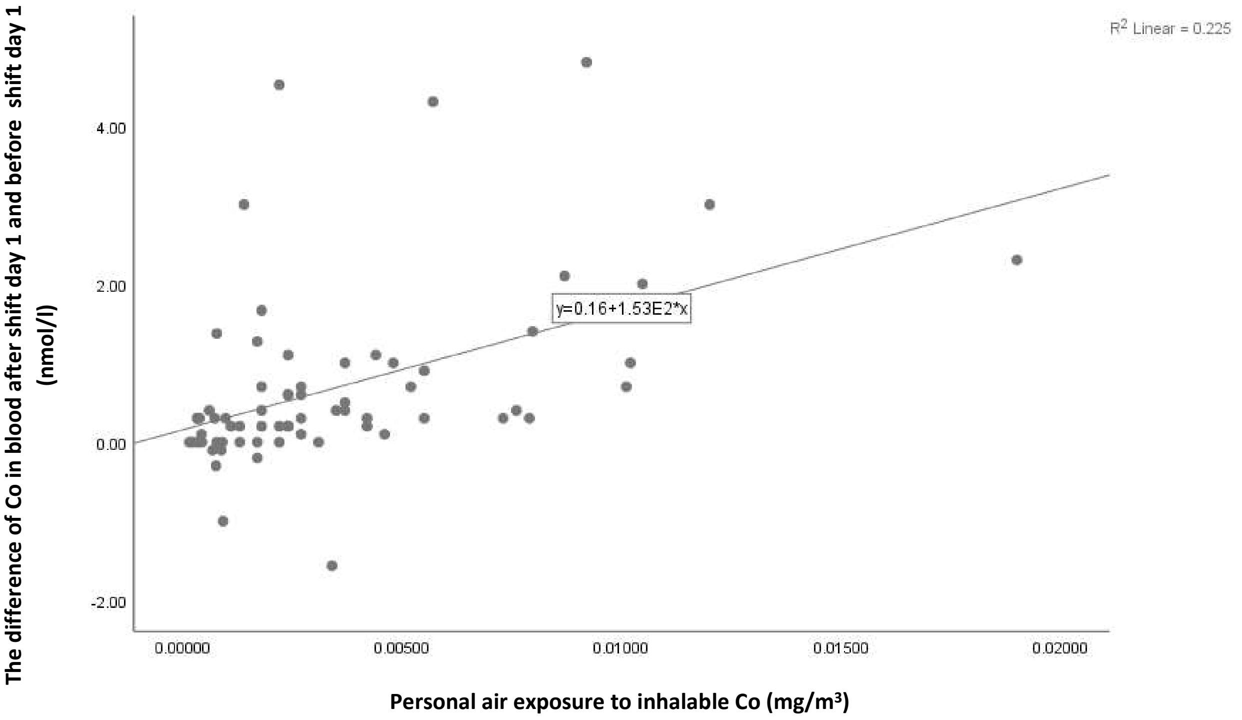

Fig 6. Scatter plot for inhalable Co compared to Co in blood.

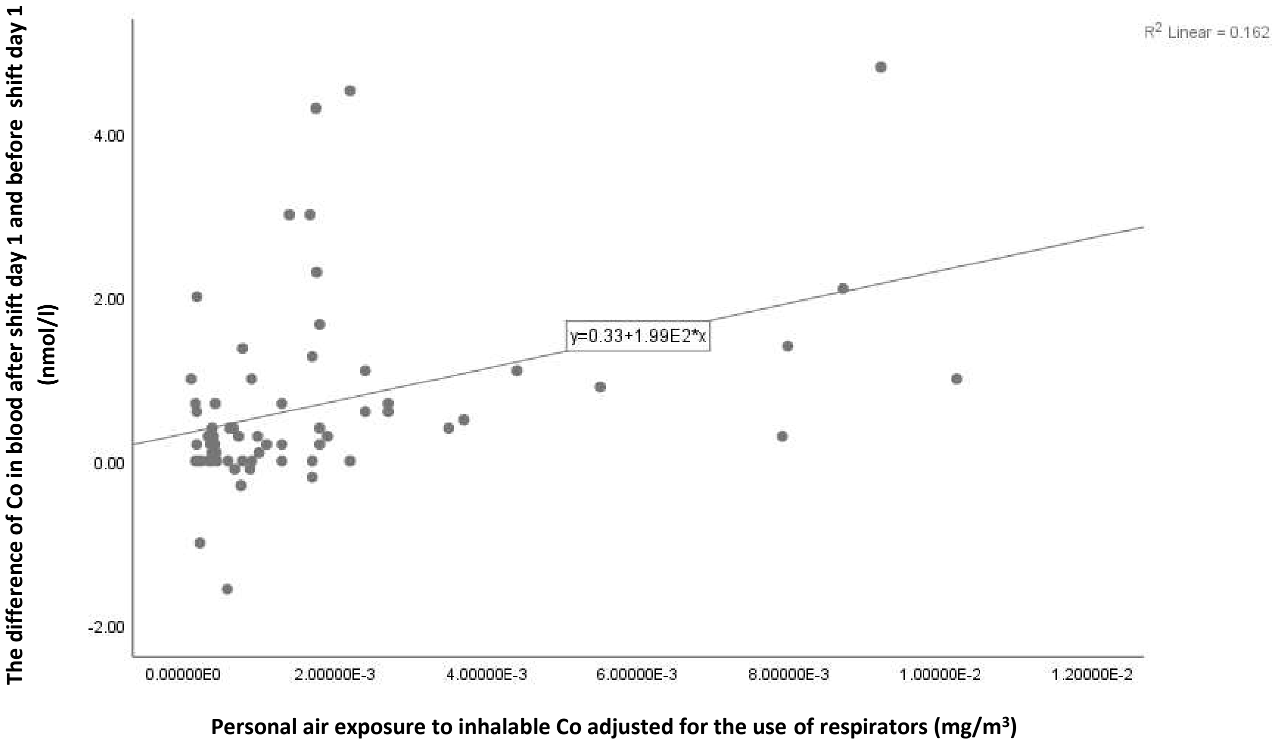

Fig 7. Scatter plot for inhalable Co adjusted for the use of respirators compared to Co in blood.

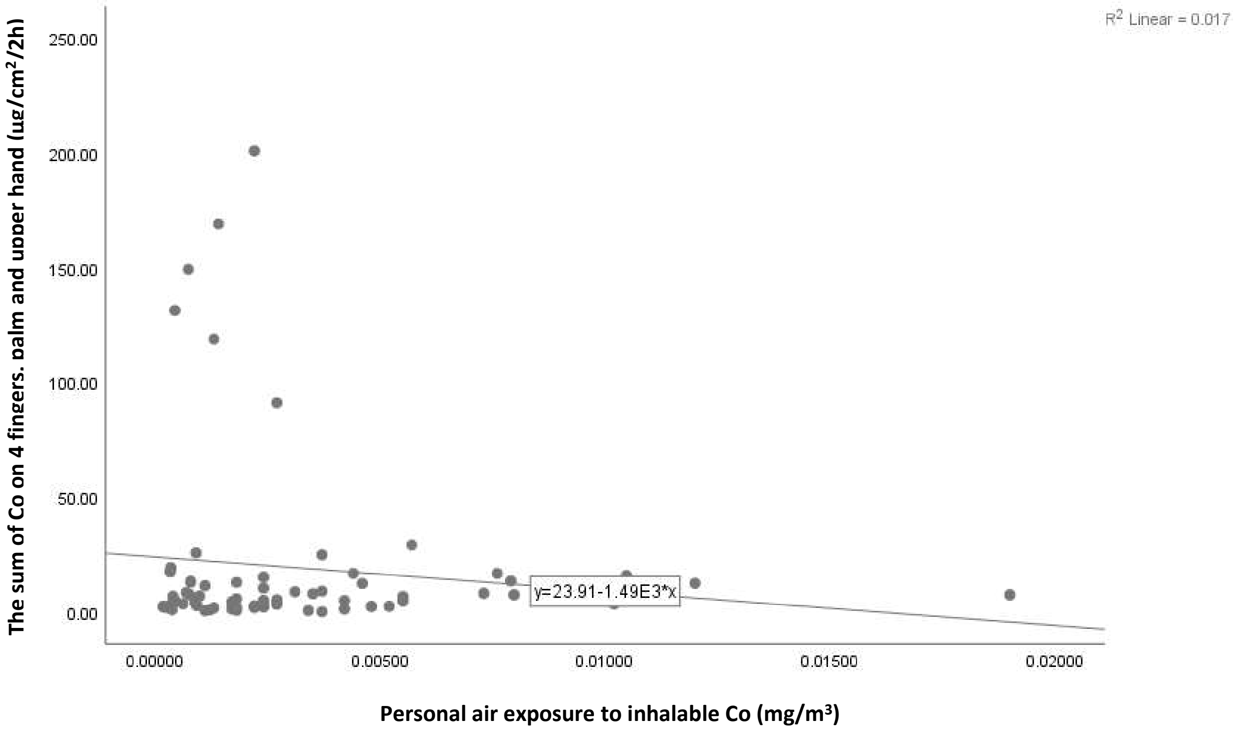

Fig 8. Scatter plot for inhalable Co compared to Co on skin.

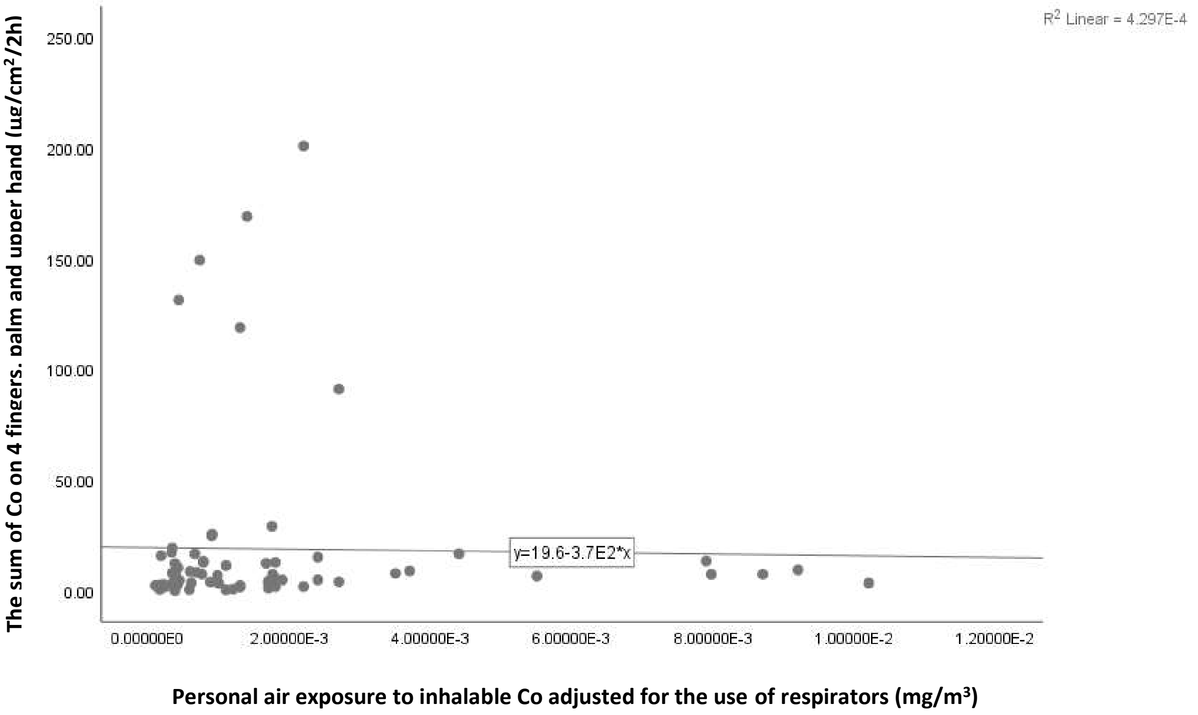

Fig 9. Scatter plot for inhalable Co adjusted for the use of respirators compared to Co on skin.
